# Comparison between automated cardiopulmonary resuscitation and manual cardiopulmonary resuscitation in the rescue of cardiac and respiratory arrest

**DOI:** 10.12669/pjms.38.8.6598

**Published:** 2022

**Authors:** Mengying Gao, Huiping Niu, Shuang Yuan

**Affiliations:** 1 Mengying Gao, Department of Emergency, The Second Hospital of Hebei Medical University, Hebei, China; 2 Huiping Niu, Department of Endoscopy Center, The Second Hospital of Hebei Medical University, Hebei, China; 3 Shuang Yuan, Department of Intensive Care Unit, Shandong Provincial Third Hospital, Cheeloo College of Medicine, Shandong University, Jinan, China

**Keywords:** Automated cardiopulmonary resuscitation, Manual cardiopulmonary resuscitation, Cardiac and respiratory arrest, Blood gas analysis index, Respiratory dynamics

## Abstract

**Objective:**

To compare the efficacy of automated cardiopulmonary resuscitation (A-CPR) and manual cardiopulmonary resuscitation (M-CPR) in the rescue of cardiac and respiratory arrest.

**Methods:**

A retrospective, single-center observational study was conducted to identify 106 patients by reviewing medical records of 269 patients with cardiac and respiratory arrest treated in The Second Hospital of Hebei Medical University, Shandong Provincial Third Hospital (Jinan, China) from February 2019 to February 2021. Patients were divided into A-CPR group (n = 55) and M-CPR group (n = 51) based on the resuscitation treatment method. The groups were matched for age, gender and the cause of cardiac arrest. Rescue effects, blood gas analysis indicators, respiratory dynamics and condition improvement of the two groups were compared.

**Results:**

In terms of rescue effects, return of spontaneous circulation (ROSC) rate, successful rate of cardiopulmonary resuscitation (CPR), 24-hour survival rate and survival discharge rate in the A-CPR group were higher than M-CPR group (*P*<0.05). With respect to blood gas analysis indicators and respiratory dynamics, the partial pressure of carbon dioxide (PaCO_2_) in the A-CPR group was lower than M-CPR group at 15 and 30 minutes after CPR, while the partial pressure of oxygen (PaO_2_), blood oxygen saturation (SaO_2_), end expiratory carbon dioxide (PetCO_2_), coronary perfusion pressure (CPP) and mean arterial pressure (MAP) in the A-CPR group were higher than M-CPR group (*P*<0.05). In aspect of condition improvement, spontaneous breathing, heart rate, spontaneous circulation, blood pressure recovery time and CPR time in the A-CPR group were shorter than M-CPR group (*P*<0.05).

**Conclusion:**

The application effect of A-CPR in the rescue of cardiac and respiratory arrest, the improvement of blood gas analysis indexes, respiration and condition improvement are more significant than M-CPR.

## INTRODUCTION

Cardiac arrest (CA) is a leading cause of mortality and morbidity globally. It is reported that there are more than 347000 and 7000 emergency medical services (EMS) responses to out-of-hospital cardiac arrest (OHCA) cases in the United States annually in adults and children, respectively.^1^ The incidence of in-hospital cardiac arrest (IHCA) is reported to be 9.7 per 1000 adult cardiac arrests and 2.7 pediatric events per 1000 hospitalizations.^2^ Prompt provision of cardiopulmonary resuscitation (CPR) is one of the priorities of adult cardiac arrest management.^3^ CPR involves clearing patient’s respiratory tract in combination with artificial respiration and chest compressions, and then carrying out professional drug intervention to establish artificial circulation and promote the recovery of cardiac function.^4^

Manual CPR (M-CPR) is the main CPR method, achieves CPR by manual external chest compression (cardiac pump and chest pump).^5^ However, the main disadvantages of manual external chest compression include limited auxiliary ventilation effect, insufficient compression frequency and depth and easy fatigue of the treating personnel. Interruptions during the compression may lead to cessation of blood supply to cerebral artery and coronary artery. In addition, manual external chest compression may cause rib and sternum fractures and directly affect the efficiency of CPR.^6^,^7^

In recent years, automated CPR (A-CPR) are designed to improve chest compression quality, are gradually replacing manual external chest compression. A-CPR allows adjustment of depth, frequency and proportion of external chest compression according to the specific situation of the patient, and provides constant and lasting ventilation support and external chest compression.^8^

Over the past years, numbers of studies have investigated the effectiveness of A-CPR and M-CPR in OHCA patients^9^,^10^,^11^, but few in IHCA patients. Therefore, we performed an observational study to analyze the efficacy of A-CPR and M-CPR in the rescue of cardiac and respiratory arrest by comparing their rescue effect, blood gas analysis index and respiratory dynamics, and condition improvement. Our hypothesis of this study was that the application of A-CPR would bring better outcomes than M-CPR in in-hospital patients.

## METHODS

**Fig.1:**
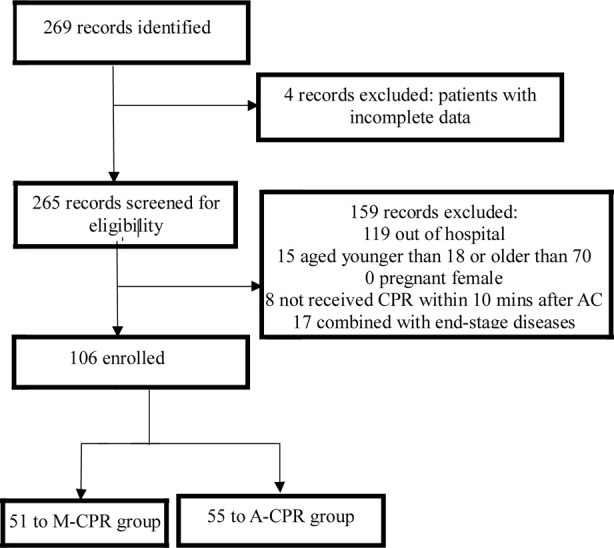
Flowchart of patient screening.

We conducted a retrospective, single-center observational study to identify 106 patients by reviewing medical records of 269 patients with cardiac and respiratory arrest who were resuscitated in The Second Hospital of Hebei Medical University, Shandong Provincial Third Hospital (Jinan, China) from February 2019 to February 2021. All the patients in the study underwent carotid artery examination. After cardiac and respiratory arrest was determined, venous access was timely established, ventilator-assisted ventilation and endotracheal intubation were performed, and adrenaline was administered repeatedly every five minutes to strengthen ECG monitoring. Patients were divided into A-CPR group (n = 55) and M-CPR group (n = 51) based on the resuscitation treatment method they received. The groups were matched for age, gender and cause of arrest (Fig.1). This study followed the Strengthening the Reporting of Observational Studies in Epidemiology (STROBE) reporting guideline.^12^

M-CPR group was performed adhering to the 2015 American Heart Association guidelines for CPR and cardiovascular first aid.^13^ Briefly, 5cm of sternal depression was located and compressions were administered at a speed of more than 100 times/minutes by 2~4 medical workers, five cycles/person, artificial respiration was performed, if the patient’s spontaneous circulation has not recovered within 30 minutes after the last spontaneous cardiac arrest, or the patient’s spontaneous circulation has successfully recovered, the rescue can be terminated.

Patients in the A-CPR group were resuscitated using SCC TM100 A-CPR (Sunlife company), connected to the air source, and the parameters were set to 30:2, resuscitation was stopped after the patient’s autonomic circulation function recovered or after 120 minutes of CPR, if the autonomic circulation function failed to recover after 120 minutes, the rescue was terminated.

### Inclusion criteria:

- Cardiac and respiratory arrest diagnosis,^14^ which was confirmed by electrocardiogram (ECG);
- Age ranges from 18 to 70 years;
- Patients received CPR within 10 mins after CA
- Patients with complete basic medical records

### Exclusion criteria:

- Death before admission;
- Pregnant women;
- Combined with end-stage diseases such as aneurysm rupture or intracerebral hemorrhage;
- Complicated with severe organ dysfunction;
- Mental disorders;
- Cardiogenic shock, heart rupture, pump failure or ventricular septal perforation before cardiac and respiratory arrest.

### Ethics Approval:

This study protocol has been approved by the hospital ethics committee (Approval number: 2022-P012, Date: 2022-03-10).

### Efficacy evaluation indicators:

Rescue effect. Briefly, return of spontaneous circulation (ROSC), successful rate of CPR, 24-hour survival rate and survival discharge rate were statistically analyzed. The recovery standard of autonomic circulation is that the pulsing of great arteries is detectable, the effective heart rates (ventricular autonomic heart rate, sinus heart rate and borderline heart rate) can be detected by ECG, the systolic blood pressure exceeds 60mmHg and lasts for three minute or more.^15^ Successful CPR was defined as ROSC maintained for more than 30 minutes.^16^

### Blood gas analysis index and respiratory dynamics:

The indexes of blood gas analysis and respiratory dynamics were measured at five, 15 and 30 minutes after CPR, patient’s venous blood samples were taken, and the oxygen partial pressure (PaO_2_), carbon dioxide partial pressure (PaCO_2_) and blood oxygen saturation (SaO_2_) were measured by Hitachi 7600 automatic biochemical analyzer. The end expiratory carbon dioxide partial pressure (PetCO_2_), coronary perfusion pressure (CPP) and mean arterial pressure (MAP) were monitored by HP 8000LED multifunctional tester.

### Condition improvement.

The patients’ spontaneous breathing, heart rate, spontaneous circulation, blood pressure recovery time and CPR time were recorded.

### Outcomes

The primary outcomes were ROSC rate and successful rate of CPR, which reflect the quality of CPR directly. The secondary outcomes included 24 hour survival rate and survival discharge rate, which may be affected by post-resuscitation care. It also included blood gas analysis indexes (PaO2, PaCO2 and SaO2) and respiratory dynamics indicators (PetCO2, CPP and MAP), and condition improvement indicators (spontaneous breathing, heart rate, spontaneous circulation, blood pressure recovery time and CPR time).

### Statistical analysis:

As per the previous study^17^ the ROSC rate of A-CPR and M-CPR were 83% and 48.8%, respectively. Based on two-tailed test and assuming power set at 0.9, and variable missing as 10%, at least 40 cases would be needed for the study^18^. SPSS 22.0 (SPSS Inc., Chicago, IL, USA) was used for data processing. Descriptive statistics were used to describe the baseline information of the patients. Continuous variables were tested for normality by Shapiro-Wilk test. Normal distributions were described as mean standard deviation and groups were compared by t-test while abnormal distributions were compared by Mann-Whitney U test. Categorical variables are described as percentage or rate and were compared by the *χ^2^* test. For the primary outcomes, adjusted odds ratios (AORs) were also calculated by multivariate logistic regression analysis with manual CPR as the reference group and controlling for the confounders (age, gender and cause of arrest). AORs were given with their 95% confidence interval (CI) and two-sided P values are presented. For the secondary outcomes, the groups were compared by the Mann-Whitney U test. *P* < 0.05 indicated that the difference was statistically significant. *P*<0.05 indicated that the difference was statistically significant.

## RESULTS

A total of 106 patients met the inclusion criteria, including 51 in the M-CPR group and 55 in the A-CPR group. As summarized in Table-I, there were 28 males and 23 females in the M-CPR group; with the age ranging from 37 to 69 years, an average of (52.53±8.40) years; among the patients, there were eight cases of poisoning, 22 cases of coronary heart disease, 12 cases of cerebrovascular disease and nine cases of other causes of cardiac and respiratory arrest. The A-CPR group included 35 males and 20 females; the age ranged from 35 to 69 years; with an average of (50.14±7.98) years; of 35 patients, there were 10 cases of poisoning, 30 cases of coronary heart disease, 10 cases of cerebrovascular disease and five cases of other causes of cardiac and respiratory arrest. There was no significant difference in general data between the two groups (*P*>0.05), as shown in Table-I.

**Table-I:** Comparison of baseline characteristics between the two groups

Characteristics	M-CPR group (n = 51)	A-CPR group (n = 55)	χ^2^/t	P
Age (mean [SD], y)	52.53±8.40	50.14±7.99	1.497	0.137
Gender (male/female)	28/23	35/20	0.837	0.360
Cause of arrest (n, %)				
Poisoning	8 (15.69)	10 (18.18)	2.630	0.452
Coronary Heart Disease	22 (43.14)	30 (54.54)		
Cerebrovascular Disease	12 (23.53)	10 (18.18)		
Other	9 (17.65)	5 (9.09)		

In terms of rescue effect, the ROSC rate [AOR = 3.61 (1.53, 8.54)], successful CPR rate [AOR = 3.11 (1.29, 7.51)], 24h survival rate [AOR = 3.65 (1.39, 9.61)] and survival discharge rate [AOR = 3.35 (1.17, 9.56)] of A-CPR group were more time three times higher than the A-CPR, and these findings were statistically significant (p<0.05), as shown in Table-II.

**Table-II:** Comparison of rescue effect between the two groups

Groups	ROSC rate		Successful CPR rate		24h survival rate		Survival and discharge rate	

	AOR(95%CI)	P	AOR(95%CI)	P	AOR(95%CI)	P	AOR(95%CI)	P

M-CPR	1 [reference]		1 [reference]		1 [reference]		1 [reference]	
A-CPR	3.61(1.53, 8.54)	0.003	3.11(1.29, 7.51)	0.012	3.65(1.39, 9.61)	0.009	3.35(1.17, 9.56)	0.024

In terms of blood gas analysis indexes and respiratory dynamics, there was no significant difference in PaCO_2_, PaO_2_, SaO_2_, PetCO_2_, CPP and MAP between the two groups at five minutes after CPR (*P*>0.05). At 15 minutes and 30 minutes after CPR, PaCO_2_ in the A-CPR group was lower than M-CPR group, while PaO_2_, SaO_2_, PetCO_2_, CPP and MAP in the A-CPR group were higher than M-CPR group (*P*<0.05), Table-III.

**Table-III:** Comparison of blood gas indicators and condition improvement between the two groups.

Outcomes	M-CPR group (n = 51)	A-CPR group (n = 55)	Mann-Whitney U test	P
Blood gas analysis indexes and respiratory dynamics				
5 mins after CPR				
PaCO_2_ (kPa)	8.89±1.27	8.78±1.49	1325.500	0.626
PaO_2_ (kPa)	4.65±1.06	4.50±1.15	1275.500	0.421
SaO_2_ (%)	66.33±8.30	64.31±7.45	1213.000	0.230
PetCO_2_ (mmHg)	14.86±1.94	14.69±1.72	1328.000	0.634
CPP (mmHg)	20.04±3.03	19.78±3.09	1325.500	0.624
MAP (mmHg)	35.15±3.18	34.63±4.25	1273.500	0.412
15 mins after CPR				
PaCO_2_ (kPa)	7.67±1.23	6.16±1.36	610.000	<0.001
PaO_2_ (kPa)	8.03±1.21	9.93±1.28	462.000	<0.001
SaO_2_ (%)	82.78±9.02	91.45±8.17	662.000	<0.001
PetCO_2_ (mmHg)	17.84±2.32	33.52±2.55	0.000	<0.001
CPP (mmHg)	18.58±2.89	38.51±3.44	0.000	<0.001
MAP (mmHg)	35.94±3.67	57.89±3.70	0.000	<0.001
30 mins after CPR				
PaCO_2_ (kPa)	6.17±1.18	3.65±1.33	254.000	<0.001
PaO_2_ (kPa)	8.66±1.22	12.39±1.34	27.000	<0.001
SaO_2_ (%)	88.76±9.25	96.83±8.34	707.000	<0.001
PetCO_2_ (mmHg)	17.02±2.63	35.65±2.94	0.000	<0.001
CPP (mmHg)	19.33±3.38	39.92±3.62	0.000	<0.001
MAP (mmHg)	37.19±3.83	59.14±3.51	0.000	<0.001
Improvement effect				
Spontaneous breathing recovery time	22.68±3.77	14.92±2.29	97.500	<0.001
Heart rate recovery time	16.78±2.94	10.76±1.91	132.500	<0.001
Spontaneous circulation recovery time	47.58±4.46	34.82±3.01	19.500	<0.001
Blood pressure recovery time	61.80±5.19	46.16±3.46	12.500	<0.001
CPR time	33.53±4.66	23.82±2.90	120.000	<0.001

In terms of condition improvement effect, the spontaneous breathing, heart rate, spontaneous circulation, blood pressure recovery time and CPR time of the A-CPR group after treatment were shorter than M-CPR group (*P*<0.05), as shown in Table-III.

## DISCUSSION

Studies have shown that A-CPR was superior to M-CPR.^8^,^17^,^19^ However, a meta-analysis of nine prospective studies has suggested that mechanical CPR was inferior to manual CPR in terms of attaining ROSC, and no differences in survival to discharge for in-hospital cardiac arrest patients.^11^ Khan et al. also reported that manual CPR is more effective in improving hospital discharge or survival at 30 days compared with mechanical CPR.^20^

In our study, the ROSC rate, successful rate of CPR, 24h survival rate and survival discharge rate of A-CPR group were more time three times higher than the A-CPR, and these findings were statistically significant (p<0.05). Our results are in agreement with the previous study of Chen YS et al.^19^ The disparity between the outcomes of the studies is thought to be that tt is hard to keep high quality M-CPR as it may be affected by fatigue after 2~3 mins of CPR.^21^ During M-CPR the blood in the right ventricle will enter the pulmonary artery, and the blood in the left ventricle will flow to the whole body. If the pressure continues to be insufficient or the pressure is withdrawn, there will be a negative pressure suction state in the chest, resulting in cardiac perfusion and blood reflux.^22^ In contrast, using A-CPR ensures that the frequency, depth and strength of extrathoracic cardiac compression are reasonably set according to the specific conditions of patients, while ensuring that the compression is maintained in a constant state, through artificial ventilation. The proportion and frequency of extrathoracic cardiac compression are accurately preset to provide patients with constant and lasting ventilation support, maintain the diastolic and systolic time ratio of 1:1, improve the quality of oxygen supply and blood supply, help to improve the rescue effect.^23^-^25^

Several studies have shown that the use of CPR auxiliary mechanical equipment not only improved the effectiveness of chest compression, but also improved hemodynamics and short-term survival.^26^,^27^ Our study demonstrated that at 15 and 30 minutes after CPR, PaCO_2_ in the A-CPR group was lower than M-CPR group, while PaO_2_, SaO_2_, PetCO_2_, CPP and MAP were higher than M-CPR group (*P*<0.05), indicating A-CPR was more efficient in improving blood gas analysis indexes and respiratory dynamics compared with the M-CPR, these results are consistent with the research results of Zhang C et al.^28^

In this study, the spontaneous breathing, heart rate, spontaneous circulation, blood pressure recovery time and CPR time of the A-CPR group after treatment were shorter than M-CPR group (*P*<0.05), indicating that the application effect of A-CPR rescue in improving the condition is also better than M-CPR. The use of A-CPR, therefore, may prevent continuous loss of brain and heart function and irreversible death by timely supplying blood to the patient’s brain and heart, and create more opportunities and favorable conditions for later defibrillation and drug treatment. This will further improve the rescue effect and the prognosis, promote the remission of the disease, and shorten the time of recovery of spontaneous breathing, heart rate, spontaneous circulation, blood pressure and CPR.^29^ Even though the A-CPR increases the diastolic blood pressure and improve the physiologic status, it does not provide strong evidence that A-CPR is better than M-CPR because systematic post-cardiac arrest care after ROSC also an impact on patient survival.^30^

### Limitation of the study:

First, this a retrospective, single center observational study without randomization. It includes a small number of cases which increased the chance of assuming false promises to be true.^31^ Improved designs with large-scale samples are needed in future studies. Second, even though age, gender and cause of arrest are controlled in our study, other confounders like BMI and health status may also influence the outcomes. Third, since post-cardiac arrest may affect patient survival, post-cardiac arrest care for the patients was not matched and analyzed in this study.^30^ Fourth, the patients included in this study were only emergency patients in our hospital. The treatment level and staffing can only partially reflect the overall treatment level of the emergency medicine department of the country. Whether this conclusion can be extended to other emergency medicine units still needs further research.

## CONCLUSION

The application effect of A-CPR in the rescue of cardiac and respiratory arrest, the improvement of blood gas analysis indexes, respiration and condition improvement are more significant than M-CPR.

### Authors’ contributions:

**HN** conceived and designed the study. **MG and SY** collected the data and performed the analysis. **HN** was involved in the writing of the manuscript and is accountable for the integrity of the study. All authors have read and approved the final manuscript.
